# A Critical Inquiry into ‘Abuse’ in EU Competition Law

**DOI:** 10.1093/ojls/gqae008

**Published:** 2024-03-07

**Authors:** Pınar Akman

**Keywords:** abuse of a dominant position, EU competition law, digital markets, more economic approach, article 102 TFEU, Court of Justice of the European Union

## Abstract

Disagreement abounds on what exactly constitutes an ‘abuse’ within article 102 TFEU, EU competition law’s prohibition of an abuse of a dominant position. This situation is highly undesirable, given the important role this prohibition is expected to play in alleviating concerns about substantial market power and its use in important sectors, typified by actions against ‘Big Tech’. This article responds to this problem by analysing and synthesising the jurisprudence of the Court of Justice of the European Union and its evolution to establish the constituent elements of an exclusionary ‘abuse’. The article corrects crucial legal misconceptions, including the putative dichotomy between violations ‘by object’ and ‘by effect’; the assumed disparate legal tests for pricing and non-pricing conduct; and the lost distinction between the ‘as efficient competitor test’ and the ‘as efficient competitor standard’. This critical inquiry allows one to draw concrete predictions on the future development of this challenging, yet vital, area of law.

## 1. Introduction

After decades in the wilderness, competition (antitrust) law is making headlines by motivating US presidential campaigns and executive orders, and being used to grill publicly the CEOs of the world’s most powerful companies.^1^ Competition law is recognised as a powerful tool to shape markets, driven by concerns about rising market concentration in the economy, typified by technology markets.^2^ Globally, an avalanche of policy proposals regarding digital markets has emerged to ‘tame’ the power of ‘Big Tech’, including through *ex ante* regulations for ‘gatekeepers’.^3^ In Europe, the prohibition of an abuse of a dominant position, found in article 102 of the Treaty on the Functioning of European Union (TFEU) (article 102) in EU competition law, takes centre stage in the application of competition law in digital markets.^4^

Digital markets are only one example demonstrating the relevance and significance of the prohibition against abuse of dominance. That prohibition applies to undertakings in all sectors of the economy, as the primary means of controlling the use of market power, to preserve effective competition. Despite the importance of the said prohibition for competitive markets and thriving economies, what constitutes an ‘abuse’ of a dominant position in EU competition law is both controversial and contested. Unfortunately, ‘the definition of an abuse in the … decisional practice and case law is imprecise’ and lacks ‘a clear normative concept capable of satisfying the basic requirements of the rule of law and legal certainty’.^5^ Moreover, ‘formulating rules which allow conduct that is harmful to competition, and therefore abusive, to be clearly distinguished is neither easy nor intuitive’.^6^ Further, the ‘lack of coherence in Article [102] case law’ leads to inconsistency within the provision and across the EU antitrust rules.^7^

Against that background, this article aims to establish the legal requirements of an abuse of a dominant position in EU competition law. This is a fundamental inquiry into the legal requisites of determining when a given practice by an undertaking with significant market power violates EU competition law. Much ink has been spilled over identifying and articulating the constituent elements of an abuse of a dominant position.^8^ Yet, no consensus or resolution has been reached on the topic in the literature or in jurisprudence, despite the central role which the prohibition of an abuse of a dominant position plays in competition law. This state of affairs generates legal uncertainty and engenders protracted litigation on fundamental legal questions, such as what *legally* constitutes an abuse.

A critical inquiry into the legally constituent elements of abuse is important, timely, and necessary. This is because the jurisprudence of the Court of Justice of the European Union (CJEU) has significantly evolved over the last decade epitomised by the Court of Justice’s (CoJ) *Intel* ruling.^9^ As the requirements of abuse are not articulated in article 102, the CJEU’s interpretation of that provision determines the necessary legal elements which establish a violation of the rule. The current article thus advances the literature by critically analysing the development and evolution of the CJEU’s jurisprudence on abuse and establishing the relevant legal requirements of ‘abuse’. The article makes an important contribution because, by explicating the legal requirements of ‘abuse’, it illuminates the correct application of article 102, which constitutes a significant portion of EU competition law and its enforcement. The article thereby also furthers the literature by revealing and correcting crucial misconceptions and oversights. Specifically, these concern: the alleged presence of an ‘object’ and ‘effect’ dichotomy under article 102; the distinction between the ‘as efficient competitor *test*’ (AEC test) and the ‘as efficient competitor *standard*’ (AEC standard); and the relation between the AEC test and a ‘more economic approach’. Furthermore, this article develops the literature by identifying the direction of the evolution of the jurisprudence on article 102 and articulating the implications of this evolution for future cases, including those in digital markets. These are timely contributions not least because the European Commission (the Commission) itself has recently embarked on a process through which it intends to adopt guidelines in order to provide ‘greater legal certainty for businesses and help foster more consistent enforcement’ of article 102, given ‘the significant developments in the case law of the [CJEU] on Article 102’.^10^ Thus, a robust understanding of the evolution of the CJEU’s case law on article 102, which this article provides, is essential for the progress of the law in this area since the prospective guidelines can only provide legal certainty and consistency in enforcement if they accurately reflect that case law.

Although article 102 does not distinguish between different categories of abuse, in practice, a distinction exists between ‘exclusionary’ abuse and ‘exploitative’ abuse. Exploitative abuse concerns conduct by a dominant undertaking that directly harms its *customers* through, for example, higher prices or reduced quality.^11^ According to the Commission, exclusionary abuse involves conduct whereby a dominant undertaking impairs effective competition in a market by foreclosing its *competitors* ‘in an anti-competitive way’, thereby adversely affecting consumer welfare.^12^ The enforcement of article 102 by the Commission has mostly involved exclusionary abuse. Due to the significance of the concept in the decisional practice, the current article likewise focuses on exclusionary abuse. A further distinction that does not exist in article 102 but has been introduced in policy documents and jurisprudence is that of price-based and non-price-based abuse, which revolves around whether the conduct involves pricing behaviour (eg rebates and discounts) or other behaviour (eg refusal to deal).^13^ This distinction has created an unnecessary and unhelpful demarcation between the putative legal tests for price-based and non-price-based abuse. This article evaluates the implications of the evolution of the jurisprudence on this distinction to identify a *uniform* test of abuse for exclusionary conduct.

This article proceeds as follows. First, it provides context to the relevance and importance of the requirements of abuse under article 102 by presenting the debate over the interpretation of that legal provision. It then examines the evolution of the jurisprudence through an identification of different eras in the case law. It analyses, first, the ‘traditional’ era of the CJEU’s case law and elaborates on the legal requirements of abuse in that era—to the extent that these are discernible—to implicate the subsequent evolution of the jurisprudence. Next, the said evolution is depicted by analysing the ‘modern’ era of the jurisprudence to establish how the legal requirements of abuse have evolved. Finally, the article identifies a ‘hybrid’ era and outlines the implications of this evolution for future enforcement practice, as well as the broader competition landscape which includes novel and untested regulatory instruments, such as the Digital Markets Act (DMA).^14^ In doing so, this article furthers the literature by establishing the legal requirements of ‘abuse’ through a critical assessment and synthesis of the jurisprudence, correcting fundamental misconceptions and oversights about the concept of abuse and the approach thereto, and prognosticating the development of the law. Consequently, it advances our understanding of a vitally important and challenging, yet widely disagreed upon, area of law.

## 2. The Debate over the Correct Approach to Article 102

Under article 102, ‘Any abuse by one or more undertakings of a dominant position within the internal market or in a substantial part of it shall be prohibited … in so far as it may affect trade between Member States’.^15^ There is no explicit defence in article 102, but it is settled law that an undertaking in a dominant position can advance an ‘objective justification’ for its conduct which, if accepted, would avoid a violation of the prohibition.^16^

In the early years of enforcement, EU competition law was criticised for adopting too formalistic an approach, which led to ‘protecting competitors’ rather than ‘protecting competition’, and for being insufficiently grounded in economics.^17^ Responding to such criticisms, the Commission pursued a ‘modernisation’ of competition law, starting in the 1990s. That modernisation encompassed all three pillars of the law, namely agreements, abuse of a dominant position and merger control.^18^ Specifically regarding article 102, following years of criticism of its form-based approach, the Commission undertook a ‘reform’ of its enforcement approach. That process aimed to align the Commission’s approach with a ‘more economic approach’ by discarding the formalistic approach in favour of one more consistent with modern economics. The reform process, formally initiated by a DG Competition Discussion Paper, culminated in the Commission guidance on article 102 in 2009.^19^ Although the ‘more economic approach’ lacks a single definition upon which a consensus exists,^20^ for current purposes, it refers to the general trend in the EU of adopting economic analysis to assess the actual and/or potential *effects* of a conduct under scrutiny for a finding that the conduct is anti-competitive.^21^

The Commission’s efforts at clarifying its enforcement principles did not end the debate surrounding article 102.^22^ Of the three pillars of EU competition law, the enforcement of the prohibition of an abuse of a dominant position has consistently been the most controversial strand. The reasons for this include: the difficulty of establishing the correct legal and economic standards for what makes *unilateral* conduct anti-competitive (as opposed to multilateral conduct such as cartels which are inherently more objectionable);^23^ the uncertainty and lack of consensus regarding the *objectives* of the prohibition of unilateral conduct; the extent to which *effects* on competitors and/or consumers and/or competition are relevant and/or necessary to prove an ‘abuse’; and the absence of commonly accepted legal and economic principles applicable to unilateral conduct.

The difficulty of establishing the parameters of abuse is aggravated by the fact that neither article 102 nor any other TFEU provision stipulates the goals of the said provision or of EU competition law rules.^24^ Protocol 27 specifies that the internal market (which the EU is to establish) includes ‘a system ensuring that competition is not distorted’.^25^ Accordingly, the purpose of EU competition law can be said to be ensuring that competition in the internal market is not distorted. Yet, this begs the question of what it means for competition to be *distorted* and how to determine that competition has been distorted on the facts. That question begs the further question of what the legal requirements of a violation are, which brings the inquiry back round to the start. Under such circumstances, the CJEU’s case law on article 102 becomes the central source of that provision’s interpretation, and it is the evolution of that jurisprudence to which this article turns next.

## 3. The Evolution of the Case Law on Abuse of a Dominant Position—Opposites Attract?

The CJEU’s antitrust jurisprudence has traditionally been viewed as formalistic and disconnected from modern economics.^26^ What was, however, unknown when the Commission embarked on its ‘reform’ to adopt a more economic approach was how the CJEU would react to that move away from a formalistic approach. The ultimate question was whether the CJEU would endorse or reject an economic, effects-based approach. Given that much of the case law is generated through appeals against Commission decisions under article 263 TFEU, and *assuming* that the Commission adopted in practice the more economic approach which it promulgated, endorsing that approach would have required a substantial change in the CJEU’s precedent on article 102. It is this change, undoubtedly, through which the law on article 102 has evolved to adopt a more economic, effects-based approach. Strikingly though, this evolution has been through not wins but losses for the Commission and/or the Legal Service on appeals, as the assumption above did not necessarily hold.^27^ It is the CJEU which has actively—and perhaps *despite* the Commission and/or the Legal Service^28^—reformed its jurisprudence to adopt an effects-based approach.

The evolution of the case law is not linear: although one can distinguish between a ‘traditional’ and a ‘modern’ era in the Court’s jurisprudence based on the *degree* to which the Court embraces or moves away from a formalistic approach, one cannot argue that one era starts where the other finishes. This is because the Court’s jurisprudence evolves by clinging to certain ‘legacy’ concepts and propositions from the traditional era whilst also incorporating elements of a more effects-based, economic approach. In a given period, the Court can deliver judgments which are more closely aligned with a formalistic or effects-based approach. This phenomenon appears particularly acute when one compares preliminary rulings with substantive judgments, where the former more willingly adopt a more economic, effects-based approach than the latter.^29^ Further, even in the same judgment, aspects of the Court’s holdings may signify a more or less effects-based approach. Thus, identifying the evolution of the case law involves establishing the *preponderance* of the more or less formalistic approach by determining the dominant trend in the jurisprudence at a given time. It is through an examination of this evolution of the jurisprudence on article 102 from the ‘traditional’ era to the ‘modern’ era that the legal requirements of establishing an ‘abuse’ can be identified. This examination is conducted next.

### A. The ‘Traditional’ Era

The TFEU does not define the concept of ‘abuse’. Subsequently, the jurisprudence of the CJEU—and particularly, of the CoJ as the final arbiter of EU law—becomes *the* legal interpretation of ‘abuse’ when establishing the parameters of conduct which infringes article 102. The CoJ’s early case law adopted a predominantly formalistic approach similar to the Commission’s pre-modernisation approach by upholding the Commission’s formalistic decisional practice in virtually all appeal cases under article 102.^30^

The CoJ’s approach in the traditional era did not adopt economics or economic methods to any significant degree. For example, aiming to establish the principles of a general concept of abuse in its seminal *Hoffmann-La Roche* ruling, the CoJ held that the concept of ‘abuse’ relates to behaviour

which is such as to influence the structure of the market where, as a result of the very presence of the undertaking in question, the degree of competition is weakened and which, through recourse to methods different from those which condition normal competition in products or services on the basis of the transactions of commercial operators, has the effect of hindering the maintenance of the degree of competition still existing on the market or the growth of such competition.^31^

This conceptualisation of ‘abuse’ is not grounded in economics and begs the question of what ‘normal competition’ means—a term which is *central* to the concept of abuse as described, but which the jurisprudence does *not* define.^32^ Indeed, if ‘normal competition’ could be defined, there would be no need separately to define abuse. Likewise, when ‘normal competition’ is not defined, the *Hoffmann-La Roche* definition of abuse simply generates the same question which it seeks to answer, namely what ‘abuse’ means. Another common, non-economic expression used by the CJEU to communicate the same notion as ‘normal competition’ is that of ‘competition on the merits’, but ‘competition on the merits’ also begs further definition and interpretation.^33^ As noted by Advocate General (AG) Rantos, the concept is ‘abstract … and cannot be defined in such a way as to make it possible to determine in advance whether or not particular conduct comes within the scope of such competition’.^34^ Thus, neither the concept of ‘normal competition’ nor that of ‘competition on the merits’—with reference to which the concept of abuse is repeatedly defined and explained in the traditional era—provides a useful test to establish whether a practice constitutes an ‘abuse’. Indeed, this ‘plethora of expressions is indicative of the objective difficulty of establishing what constitutes abusive conduct’.^35^

In its formational years, the CoJ’s jurisprudence bestowed upon dominant undertakings a so-called ‘special responsibility’ not to allow their behaviour to impair genuine, undistorted competition in the internal market.^36^ Similar to ‘normal competition’ and ‘competition on the merits’, the concept of special responsibility does *not* exist in the Treaty and is *not* defined by the courts, despite its becoming a mantra for the courts.^37^ The explanation provided in the jurisprudence for special responsibility—another non-economic concept—states the obvious position that a dominant undertaking should not impair undistorted competition through its conduct.^38^ This explanation states the obvious because article 102 explicitly imposes a legal obligation on dominant undertakings by *prohibiting* the abuse of a dominant position—an obligation which is not imposed on any undertaking which is not dominant. Consequently, the concept of ‘special responsibility’ adds nothing to further distinguish or qualify that legal obligation imposed on dominant undertakings by article 102 *not to abuse* their dominant position. The concept of special responsibility does not illuminate which practices may constitute an abuse. Thus, for the purposes of legally establishing which conduct violates the prohibition and on what basis, ‘special responsibility’ provides no advancement either.

The Court’s traditional era approach to article 102 was particularly unclear regarding whether evidence of the anti-competitive *effects* of a practice was necessary to establish an abuse or whether an abuse could be found based on the *form* of the practice. This unclarity is demonstrated by virtue of some judgments which require an assessment of effects of conduct and others which do not.^39^ Indeed, based on this, some commentators argue that an object–effect dichotomy has been introduced into the case law on article 102, similar to the distinction in article 101 TFEU (article 101).^40^

In some of its later jurisprudence in this era, eg in *Deutsche Telekom I* and *TeliaSonera*, the Court stipulated that, for conduct to constitute an abuse, the contested practice must have an *effect* on equally efficient competitors whereby their access to or growth in the market is restricted or prevented, and that the practice in question (margin squeeze) cannot be found to constitute an abuse without demonstrating an anti-competitive *effect*.^41^ Likewise, in *Bronner*, the CoJ emphasised that for the contested conduct (refusal to deal) to constitute an abuse, it had to be demonstrated that the input sought from the dominant undertaking was indispensable and the refusal to provide access to it would *eliminate all competition* from the undertaking requesting access.^42^ Thus, an assessment of the effects of such a refusal on the competition from the competitor seeking access was required before identifying an abuse.

Yet, in other cases, the Court did not require a demonstration of effects in expressing the requirements of abuse. In such cases, the Court’s approach was based largely on formalistic reasoning to the exclusion of fundamental economic parameters such as price–cost analysis, market coverage of the contested practice and effects on competition (as opposed to competitors).^43^ For example, in *AKZO*, the Court held that prices below average variable costs by means of which a dominant undertaking seeks to eliminate a competitor ‘must be regarded as abusive’ without the need to demonstrate, for example, that the dominant undertaking may be able to recoup its losses after the predation period.^44^ Similarly, in *Michelin I*, regarding rebates, the Court focused the test of abuse on whether

the discount tends to remove or restrict the buyer’s freedom to choose his sources of supply, to bar competitors from access to the market, to apply dissimilar conditions to equivalent transactions with other trading parties or to strengthen the dominant position by distorting competition.

In doing so, it emphasised the freedom of competitors and customers.^45^ Likewise, in *British Airways*, the Court expressly held that ‘In the *Hoffmann-La Roche* and *Michelin* judgments, the [CoJ] found that certain discounts granted by … undertakings in a dominant position were abusive in character’.^46^ This is perhaps the closest the Court came to expressing that certain practices by dominant undertakings are presumptively (ie by-object) abusive. However, the Court did *not* use any language to remark that these practices are abusive *by object* even though such language was integral to the Court’s lexicon given the concept’s centrality in article 101. Furthermore, as discussed later, it is precisely this line of case law which the Court overturned in *Intel*.^47^

For the sake of completeness—even if pronouncements of the law by the General Court (GC) are not on par with those of the CoJ—it is worth discussing the GC’s ruling in *Michelin II*, which does directly refer to ‘object’ and ‘effect’ under article 102. In *Michelin II*, the GC held that, for the purposes of applying article 102,

establishing the anti-competitive object and the anti-competitive effect are one and the same thing. If it is shown that the object pursued by the conduct of an undertaking in a dominant position is to limit competition, that conduct will also be liable to have such an effect.^48^

This statement requires further discussion. This is because the GC appears to articulate the exact opposite elsewhere in *Michelin II*.^49^ Moreover, the GC’s statement equating anti-competitive object and effect under article 102 is made with reference to *Irish Sugar*, where the GC’s position appears also to suggest the *opposite* of what the GC (re)iterated in *Michelin II*. In *Irish Sugar* (in the paragraph which is the source reference for the statement in *Michelin II*), the GC held that

the arguments which the applicant draws from the confusion between the object and the effect of the practice in question must be rejected, since, as the Commission emphasises, Article [102] does not distinguish between the object and the effect and reference is made both to the anti-competitive object and to the anti-competitive effect of that practice in the contested decision.^50^

Thus, the GC did *not* hold that anti-competitive object and anti-competitive effect are one and the same thing under article 102 in *Irish Sugar*—rather, the Court explicitly stated that this distinction does *not exist* in article 102.^51^

To be clear, the GC’s holding in *Irish Sugar* does not mean that the object pursued by or the effects of the conduct are irrelevant to assessing abuse. What the Court appears to have stipulated (correctly) in *Irish Sugar* is that the *dichotomy* found in article 101, where a practice may be found to constitute a violation purely by virtue of its object (to distort, prevent or restrict competition) or by virtue of its effects (which would *only* be relevant to assessing those practices which are not by-object violations), does not exist under article 102.

That there is no dichotomy of object and effect under article 102 is clearly supported by the text of the TFEU itself, which does not include such a distinction in article 102. In fact, such a dichotomy cannot be read into article 102 without infringing basic principles of statutory interpretation: as the legislator has specifically included that distinction in article 101, the absence of the distinction in article 102 must be read as a refusal through statutory silence by the legislator.^52^ This position is also confirmed in the modern era judgments of the CJEU, as discussed below.^53^

The implication of the absence of a dichotomy between by-object and by-effect violations under article 102 for the purposes of establishing the legal requirements of abuse is that the CoJ can (re)formulate its jurisprudence to adopt and endorse a more (or less) effects-based approach. The absence of the dichotomy further means that the evolution of the jurisprudence can be progressively holistic because there does not have to be, as a matter of primary law, a residual by-object category of violations under article 102.^54^ Thus, the CoJ can abandon its formalistic approach completely without violating the Treaty. Likewise, the absence of the dichotomy implies that when the CoJ adopts an approach requiring an assessment of the *effects* of a given conduct on competition, consumers, etc for the conduct to constitute an abuse, it does not do so to define an approach applicable only to some practices (ie those which are not by-object violations) or to coexist alongside another (eg by-object) approach under article 102. Any new approach promulgated by the CoJ must thus be interpreted as *the* approach to abuse, subject only to any limitations that the Court itself may provide in formulating that approach. The next section discusses the ‘modern’ era of the Court’s jurisprudence to demonstrate such a progressively holistic change in the approach to the requirements of abuse under article 102.

### B. The ‘Modern’ Era

Numerous recent CoJ judgments reveal a trend towards predominantly adopting a more effects-based and less formalistic approach to article 102. The CoJ’s ‘modern’ jurisprudence can, indeed, be argued to have gone further than the Commission’s guidance and decisional practice in endorsing an economic approach to article 102, through its focus on the *effects* of conduct.^55^ The modern era started around a decade ago, and developed up to and climaxed with *Intel*, before progressing further. Thus, the following discussion is divided into the pre-*Intel* and post-*Intel* periods to illustrate the progression of the jurisprudence, which demonstrates a shift in paradigms away from the traditional, predominantly formalistic approach.

#### (i) *Pre-*Intel *and* Intel

In a line of cases starting most notably with *Post Danmark I*, the CoJ has gradually embraced a more economics-oriented approach and expressly rejected a formalistic approach which protects any and all competitors for its own sake or a particular market structure.^56^ According to the Court in *Post Danmark I*,

[c]ompetition on the merits may, by definition, lead to the departure from the market or the marginalisation of competitors that are less efficient and so less attractive to consumers from the point of view of, among other things, price, choice, quality or innovation.^57^

Further, the Court held that article 102

prohibits a dominant undertaking from, among other things, adopting pricing practices that have an exclusionary effect on competitors considered to be as efficient as it is itself and strengthening its dominant position by using methods other than those that are part of competition on the merits.^58^

The Court promulgated the position adopted in *Post Danmark I* and established the necessity of anti-competitive *effects* in abuse of dominance cases in several subsequent judgments. A particularly important statement, expressed in numerous rulings, is that ‘Article [102] prohibits a dominant undertaking from, inter alia, adopting pricing practices which have an exclusionary effect on its equally efficient actual or potential competitors’.^59^ Although the necessary anti-competitive effects do not have to be actual effects for the conduct to constitute abuse, *at a minimum*, a demonstration of ‘potential’ or ‘likely’ anti-competitive effects is necessary for a practice to be unlawful under article 102.^60^ This means that such effects ‘must not be of purely hypothetical’ nature or speculative.^61^ In thus embracing an economic, effects-based approach, the Court has adopted the AEC *standard* (not to be confused with the AEC *test*)^62^ as the standard for abuse under article 102, given the Court’s indifference to the exclusion or marginalisation of competitors which are less efficient than the dominant undertaking from the market. The CoJ has similarly confirmed that conduct cannot be found to constitute an abuse without an assessment of its exclusionary *effects* on competition coming from equally efficient actual or potential competitors of the dominant undertaking. Thus, a practice cannot constitute an abuse based merely on its form or type: an assessment of its effects on competitors as efficient as the dominant undertaking needs to be undertaken.

The momentum behind the move towards an effects-based approach in jurisprudence did not reach the point of shifting paradigms for many years. The watershed in the modern era is the Court’s ruling in *Intel*—a judgment which ‘hit the European competition law community like a bombshell’.^63^

In *Intel*, the CoJ reiterated that article 102 does not ‘seek to ensure that competitors less efficient’ than the dominant undertaking should remain on the market and ‘not every exclusionary effect is necessarily detrimental to competition’.^64^ Notably, neither of these principles is expressly limited to a particular type or category of abuse (eg price-based conduct, rebates) and both are stipulated as general principles. *Intel*’s significance lies in the fact that, therein, the CoJ—sitting as the Grand Chamber—quashed a GC judgment in which the GC had principally applied the then-settled jurisprudence of the CoJ to the facts.^65^ This is all the more remarkable since, up until *Intel*, the Commission’s record in article 102 appeals contained virtually no losses.^66^ Thus, *Intel* represents a clear turning point in the evolution of the law on the abuse of dominance.

The substantive importance of *Intel* is that in its ruling—albeit rather cryptically—the CoJ ‘clarified’ some of its most form-based precedent (on exclusivity/loyalty rebates) and held that to be abusive, conduct must be ‘capable of restricting competition’ and of ‘producing the alleged foreclosure effects’.^67^ This was expressed by the Court through a ‘rather circuitous route’.^68^ This is because the Court held that the *Hoffmann-La Roche* line of cases must be ‘further clarified in the case where the undertaking concerned submits, during the administrative procedure, on the basis of supporting evidence, that its conduct was not capable of restricting competition and, in particular, of producing the alleged foreclosure effects’.^69^ The Court then stated unambiguously that where the dominant undertaking argues during the administrative procedure (with supporting evidence) that its conduct is incapable of restricting competition and of producing foreclosure effects, the Commission ‘is required to’ analyse *all* of the following: first, the extent of the undertaking’s dominant position on the relevant market; second, the share of the market covered by the challenged practice; third, the conditions and arrangements for granting the rebates in question, their duration and their amount; and fourth, the Commission must also ‘assess the possible existence of a strategy aiming to exclude competitors that are at least as efficient as the dominant undertaking from the market’.^70^ This four-pronged assessment is an ‘analysis of the capacity to foreclose’.^71^ Thus, it is clear that the Court ‘clarified’ its traditional, highly formalistic jurisprudence on the topic to adopt an approach which analyses the *effects* of the practice through an assessment of various economic and contextual factors, including a strategy to exclude as-efficient competitors, before establishing a violation.

Two questions with broad significance were debated after *Intel*. One was whether the AEC framework only applied to price-based abuses (ie where an assessment of the effects of a dominant undertaking’s *pricing* conduct on competition was possible).^72^ The second question was whether the *Intel* approach was confined to cases when the Commission applied the AEC test, namely whether the ruling constituted merely a procedural point of law. Such an interpretation would have meant that the approach articulated in *Intel* by the CoJ was inapplicable to cases where the Commission did not apply the AEC test and/or the dominant undertaking did not contest the Commission’s application of that test.^73^ This second question arose because of the cryptic way in which the Court explained the requirement for the Commission to assess the conduct’s capacity to foreclose.^74^

One can start with the first question. Some commentators interpreted *Intel* as providing a framework applicable solely to price-based practices, since the case itself concerned pricing conduct.^75^ However, such an interpretation is unduly restrictive because the Court’s particular expression of the legal approach in *Intel* was *not* limited to any given practice, being articulated in general terms.^76^ In the absence of a limiting expression by the Court and based on a literal reading of the ruling, restricting the relevance and application of the ruling to price-based conduct appears unjustified. Indeed, the Court’s most recent jurisprudence clearly rebuts a restrictive interpretation of *Intel* as applicable only to pricing conduct (or only to rebates), as discussed below.^77^

The second question is answered to a degree through a careful reading of the judgment itself. First, in stipulating the factors which the Commission ‘is required to analyse’ to determine the conduct’s ‘capacity to foreclose’,^78^ the Court made *no* remarks to suggest that this analysis was required only in cases where the AEC *test* was relevant or necessary, or where it was used or should have been used by the Commission. The statements of the Court were of general applicability. This is because the Court further noted that the analysis of the ‘capacity to foreclose’ was relevant *also* to the assessment of any objective justification and efficiencies which may outweigh the ‘unfavourable *effects* of the practice in question on competition’.^79^ For the CoJ, that assessment of objective justification and efficiencies ‘can be carried out … *only after*’ the Commission analyses ‘the intrinsic capacity of that practice to foreclose competitors which are at least as efficient as the dominant undertaking’.^80^ Thus, the Commission cannot assess an objective justification or efficiency defence advanced by the dominant undertaking without having first assessed the conduct’s capacity to foreclose as-efficient competitors. Given that dominant undertakings can raise objective justifications and efficiency defences in *all* investigations under article 102 and that the Court makes no distinction in its statement of the connection between the assessment of objective justification and efficiencies and the capacity to foreclose, the Court’s stipulation of the correct approach must apply to *all* exclusionary abuses. Furthermore, in *Intel*, the CoJ held that the GC erred in its failure to ‘examine all of the applicant’s arguments seeking to call into question the validity of the Commission’s findings concerning the foreclosure capability of the rebate concerned’.^81^ Admittedly, the Court introduced more cryptic language into the ruling by drawing the scope of the GC’s obligation to conduct such a review to apply ‘If, in a decision finding a rebate scheme abusive, the Commission carries out such an analysis [of capacity to foreclose]’.^82^ This could be interpreted to suggest that the Commission is not obliged to analyse foreclosure capacity in all cases and if the Commission has not done so, the GC does not need to concern itself with the foreclosure capability of conduct either, since in such a case the undertaking also cannot bring forward evidence to rebut a non-existent foreclosure analysis. That said, the Court remitted the case to the GC, rather than give final judgment itself, because ‘The review by the [GC], in the light of the arguments put forward by Intel, of *whether the rebates at issue are capable of restricting competition*’ involves examining factual and economic evidence, which the GC must undertake.^83^ Thus, the Court’s phrasing of the remittal rationale suggests that the relevant legal question was not simply one of procedure (which the Court could have answered), but one of *substance*, namely that of assessing whether the contested rebates were ‘capable of restricting competition’, as a matter of fact, economics and law. The jurisprudence following *Intel* has, indeed, vindicated this reading of *Intel* and is discussed next.

#### (ii) *Post-*Intel

The paradigm shift in the jurisprudence continued in cases following *Intel*. These cases establish the true significance, relevance and application of the principles espoused in *Intel*. Both the CoJ and the GC have widely used *Intel* and the principles iterated therein as general statements of the law expressing the correct approach in subsequent article 102 cases involving practices of all types.^84^ One example is *Generics*, where the CoJ held that

if [conduct] is to be characterised as abusive, that presupposes that that conduct was capable of restricting competition and, in particular, producing the alleged exclusionary effects … *and* that assessment must be undertaken having regard to all the relevant facts surrounding that conduct.^85^

It is significant that the Court’s holding states the *general* approach to abuse, including price-based *and* non-price-based conduct. This is because, in this ruling, the Court cites jurisprudence on pricing conduct (margin squeeze and rebates) in restating the same general principle in a case involving a *non-price-based* conduct (pharmaceutical pay-for-delay agreements). Similarly, in *Slovak Telekom*, the Court explained that for practices to constitute abuse, they must be ‘able to give rise to at least potentially anti-competitive effects, or exclusionary effects, on the markets concerned’.^86^

Most recently, in *ENEL*, the Court overtly rejected a form-based approach in favour of an effects-based approach in ruling that

[g]iven that the abusive nature of a practice does not depend on the form it takes or took, but presupposes that that practice is or was capable of restricting competition and, more specifically, of producing, on implementation, the alleged exclusionary effects, that condition must be assessed having regard to all the relevant facts.^87^

The Court similarly reiterated that the relevant effects were those on competitors which were at least as efficient as the dominant undertaking and that the effects had to be more than hypothetical even though it was sufficient that the conduct was *capable* of producing such exclusionary effects.^88^

In *ENEL*, a case concerning non-pricing conduct, the Court notably confirmed that the AEC *standard* is applicable to both pricing and non-pricing conduct.^89^ The Court explicitly held that

[t]he relevance of the material or rational impossibility for a hypothetical competitor, which is as efficient but not in a dominant position, to imitate the practice in question, in order to determine whether that practice is based on means that come within the scope of competition on the merits, is clear from the case-law on practices both related and unrelated to prices.^90^

Thus, in *ENEL*, the Court gave a definitive answer in the negative to the first question debated after *Intel*, namely whether the AEC standard was relevant to only price-based conduct. Jurisprudence following *ENEL* has corroborated the Court’s position that the *Intel* principles apply to both pricing and non-pricing conduct, and that the AEC standard and an assessment of foreclosure capability are relevant to assessing both. In *Unilever*, the CoJ unambiguously confirmed that the ‘clarification’ it offered in *Intel* applied also to exclusive dealing, and thus, by implication, to non-pricing practices.^91^ Thus, the CoJ unequivocally affirmed that the framework set out in *Intel*, which requires an effects-based approach analysing the conduct’s capacity to foreclose, is applicable to *all* exclusionary abuses.^92^

It is also clear that the GC has interpreted the CoJ ruling in *Intel* as instating the *general* approach to abuse. In *Google (Search) Shopping*, the GC clarified that *Intel* had stipulated the general approach to exclusionary abuse by holding that

to find that Google had abused its dominant position, the Commission had to demonstrate the—at least potential—effects attributable to the impugned conduct of restricting or eliminating competition on the relevant markets, taking into account all the relevant circumstances, particularly in the light of the arguments advanced by Google to contest the notion that its conduct had been capable of restricting competition.^93^

In this case, the GC also confirmed the absence of an object–effect dichotomy under article 102 by ruling that ‘unlike Article [101], Article [102] does not distinguish forms of conduct that have as their object the prevention, restriction or distortion of competition from those which do not have that object but nevertheless have that effect’.^94^ The GC further inferred from this and the CoJ’s related jurisprudence regarding exclusionary abuses that

a practice cannot be categorised as abuse of a dominant position unless it is demonstrated that there is an anticompetitive effect, or at the very least a potential anticompetitive effect, although, in the absence of any effect on the competitive situation of competitors, an exclusionary practice cannot be classified as abusive vis-à-vis those competitors.^95^

The second question raised by *Intel*, namely whether *Intel* concerned the substantive approach to article 102 or merely a procedural point, is answered unambiguously by both the CoJ’s *Unilever* ruling and the GC’s *Intel RENV* ruling. In *Unilever*, the CoJ explained that *Intel* means that a competition authority ‘must ensure, at the stage of classifying the infringement, that [the relevant clauses] were … actually capable of excluding competitors as efficient as [the dominant] undertaking from the market’ where the dominant undertaking disputes that its conduct can exclude as-efficient competitors or argues that its conduct is justified.^96^ In *Intel RENV*, the GC conducted a substantive and legal assessment of the Commission’s decision following the CoJ’s *Intel* ruling and found that the Commission had failed to assess adequately the foreclosure effects of the contested conduct.^97^ This was because the Commission failed properly to examine the duration and amount of the contested rebates.^98^ Notably, this finding was reached ‘notwithstanding the conclusions which may be drawn from the [AEC] test’ and essentially *in addition* to the separate scrutiny of the Commission’s application of that test in the decision.^99^ This stance of the Court demonstrates that the CoJ’s *Intel* ruling did not simply concern a point of procedure regarding the application of the AEC test by the Commission, but rather established (or ‘clarified’) the applicable substantive law, which the GC applied in *Intel RENV*.^100^*Intel RENV*, indeed, led to the ultimate annulment of the Commission’s decision in *Intel*.^101^ The GC concluded that ‘the Commission is not in a position to determine that the applicant’s rebates and payments at issue were capable of having or likely to have anticompetitive effects and that they *therefore* constituted an infringement of Article 102 TFEU’.^102^ From this critical statement (found in the judgment’s ‘conclusion on the application for annulment of the contested decision’), it is clear that the reason for annulment concerns the *substantive* assessment of conduct and that an infringement of article 102 cannot be established without demonstrating the capability or likelihood of the conduct’s generating anti-competitive *effects*. The GC also reaffirmed in *Intel RENV—*once again—that there are no by-object restrictions under article 102 in stating that

although a system of rebates set up by an undertaking in a dominant position on the market may be characterised as a restriction of competition, since, given its nature, it may be presumed to have restrictive effects on competition, the fact remains that what is involved is … a mere presumption and not a per se infringement of Article 102 TFEU, which would relieve the Commission in all cases of the obligation to examine whether there were anticompetitive effects.^103^

This position is corroborated by the CoJ in *Unilever* holding that the ‘ability’ of exclusivity clauses—another practice arguably subject to a by-object approach—‘to exclude competitors is not automatic’, thus necessitating an analysis of their ‘capacity to foreclose’ as-efficient competitors.^104^ The recent pronouncements of the CJEU thus confirm that by-object restrictions which exist under article 101 and which do, indeed, ‘relieve the Commission in all cases of the obligation to examine whether there were anticompetitive effects’ do *not* exist under article 102.^105^

The GC’s stance in *Intel RENV* was solidified in *Qualcomm*, which unprecedentedly saw another Commission decision on article 102 quashed on appeal, within the space of six months.^106^ Interestingly, in *Qualcomm*, the Commission *had* analysed foreclosure capability, but the GC found that the analysis failed properly to account for certain facts and that the evidentiary basis for the decision was insufficient.^107^ In paraphrasing *Intel*, the GC held that ‘in the case where’ an undertaking submits evidence during the administrative procedure that its conduct was not capable of restricting competition and producing the alleged foreclosure effects, ‘it is for the Commission to carry out an analysis of the foreclosure capacity of competitors that are at least as efficient’.^108^ The GC’s holding, with respect, lacks internal coherence and is circular: it requires the undertaking to advance evidence to show that its conduct is not capable of ‘producing the alleged foreclosure effects’ as a requirement for the Commission’s conducting an analysis of the foreclosure capacity of the practice, but the undertaking cannot be in a position (or in need) of bringing evidence to show that the conduct is *in*capable of ‘producing the alleged foreclosure effects’ if the Commission has not analysed the foreclosure capacity of the conduct, which the Court obliges the Commission to undertake *after* the undertaking puts forward evidence to rebut the alleged foreclosure effects.

The GC further added that

if such conduct is to be characterised as abusive, that presupposes that that conduct was capable of restricting competition and, in particular, producing the alleged exclusionary effects, and that assessment must be undertaken having regard to all the relevant facts surrounding that conduct.^109^

This second statement is clearly made in more general terms and without qualifications pertaining to evidence advanced by a party during the investigation. Then again, the GC continued its elaborations with another ‘if’ and stipulated with reference to *Intel* that

[i]f, in a decision, the Commission carries out such an analysis, the Court must examine all of the applicant’s arguments seeking to call into question the validity of the Commission’s findings as to the foreclosure capacity of competitors that are at least as efficient …^110^

Regrettably, this part of the Court’s ruling is also internally incoherent because there is no mention of the Commission’s carrying out *any* analysis in the immediately preceding paragraph to which this statement refers and elaborates on. Thus, the two paragraphs of the judgment ([355], [356]) appear to be placed in the wrong order, which creates uncertainty regarding the Court’s position on the approach to establishing abuse.^111^ The Court, nevertheless, reiterated that there are no by-object violations under article 102 in rejecting a form-based approach which could be used to establish a violation of the provision through a formal characterisation of the relevant conduct.^112^

Besides establishing an economic, effects-based approach to article 102 and rejecting an object–effect dichotomy thereunder, judgments such as *Generics*, *Google (Search) Shopping*, *ENEL*, *Intel RENV*, *Qualcomm* and *Unilever* are notable for promulgating a uniform approach to article 102. The ‘modern’ CJEU jurisprudence demonstrates that there is a single approach applicable to *all* types of exclusionary abuse under article 102 since these cases involved both pricing and non-pricing conduct and espoused the same principles.^113^ Given the number of clarifications and confirmations which the CJEU provided in the modern era, the evolution of the jurisprudence can only be characterised as shifting paradigms in approach. The next subsection dissects this evolved approach to synthesise and clarify the legal test for exclusionary abuse.

#### (iii) *The legal test for exclusionary abuse*

*Intel* and subsequent judgments have confirmed the relevance and application of an *effects-based* approach to abuse. Current jurisprudence predominantly requires an analysis of the foreclosure effects of the conduct on (hypothetical) competitors which are at least as efficient as the dominant undertaking for a finding of exclusionary abuse.^114^ This is so for *all* types of unilateral conduct, including practices historically viewed with a high degree of competitive concern (such as exclusivity/loyalty rebates) and irrespective of whether the conduct is price-based or non-price-based. This assessment of the capacity to foreclose through anti-competitive effects must be undertaken ‘having regard to all the relevant facts’ surrounding the contested conduct.^115^ The effects do not need to be actual and can be potential effects, but must be more than speculative or hypothetical. The current state of the jurisprudence indicates (and confirms) that there are *no* presumptively unlawful practices in the sense of by-object restrictions under article 102—*all* practices must be assessed for their effects before they can be deemed abusive.^116^ Accordingly, conduct *cannot* be found to constitute an abuse based on its form alone. The overall jurisprudential trend is thus a decisive move towards adopting an effects-based approach and away from a form-based approach, by incorporating the AEC standard as the lodestar.

To understand correctly what this applicable legal standard involves, one must distinguish the AEC *standard* from the AEC *test*. This crucial distinction is overlooked in some commentary on article 102.^117^ The *standard* here can be understood as a general framework or a *principle* which determines whether a given practice is competitive or anti-competitive.^118^ The AEC standard was originally proposed by Posner as a principle to judge practices which are allegedly exclusionary and was termed ‘the equally efficient or more efficient competitor standard’.^119^ The AEC standard thus conceived involves, first, establishing monopoly power, and second, providing the possibility of rebuttal by proof that the practice is, on balance, efficient.^120^ The rationale behind the standard is that ‘The fact that a firm has monopoly power does [not] mean that the law should prevent it from competing’ and ‘It would be absurd to require the firm to hold a price umbrella over less efficient rivals’ because ‘we want to encourage efficiency’.^121^ Notably, Posner’s AEC *standard* does *not* refer to any price or cost levels. Rather, below-cost (predatory) pricing is only an *example* of a practice that might exclude an equally or more efficient firm.^122^ In contrast, when commentators and practitioners (including competition authorities such as the Commission) refer to the AEC *test*, they generally refer to a technical price–cost analysis, which determines whether the price charged by the dominant undertaking is above or below a certain level of cost and whether the ‘effective price’ against which competitors have to compete is below cost (and thus unprofitable, and potentially exclusionary).^123^ The pertinent issue is that whether a price–cost test is used in a case as the appropriate technical method is a separate point to that of which standard guides the enforcement practice. The standard remains applicable irrespective of which test is used to demonstrate whether the standard has been technically violated in a case.^124^ This distinction between the standard and the test was also overlooked in some of the GC’s recent jurisprudence.^125^ Yet, the distinction is adopted in one of the most recent CoJ rulings on article 102, namely *ENEL*.

In *ENEL*, the Court seemingly provides a systematic rationalisation of the various approaches to article 102 and, most notably, stipulates—after essentially holding that the AEC standard is relevant to all abuses^126^—that the AEC test only applies (and *must* be applied ‘as a general rule’) to pricing conduct and the test for non-pricing conduct can be found in *Bronner*.^127^ Although this is not a satisfactory resolution regarding non-pricing conduct because of the limitations of *Bronner* (a case concerning one specific type of non-pricing conduct (refusal to supply)), the Court’s pronunciation is welcome because it essentially distinguishes the AEC *standard* from the AEC *test*. The Court, indeed, goes beyond stating the relevance of the AEC standard to non-pricing conduct and establishes it as the applicable standard in the case at hand, which concerns non-pricing conduct.^128^ The CoJ thereby *implicitly* confirms the distinction between the AEC *standard* and the AEC *test* by holding that the former remains applicable even where the latter has no direct application.^129^ Consequently, the Court validates the importance and relevance of the AEC standard to the *general* approach to abuse under article 102 and a unitary standard for abuse, as herein set out, emerges.

The distinction between the AEC *standard* and the AEC *test* also explains the CoJ’s holding in *Intel* that even where a dominant undertaking’s rebates have an exclusionary effect on competitors which are ‘at least as efficient as’ the dominant undertaking, the possibility of objectively justifying the practice or demonstrating that efficiencies outweigh anti-competitive effects remains.^130^ Were the AEC *standard* and the AEC *test* the same thing, such a possibility could not exist: where the test shows that the practice can exclude as-efficient competitors, if the test and the standard are the same thing, then failing the test (eg because the effective price is below cost) would terminate the legal and economic assessment. *Because* the standard is a principle which remains applicable irrespective of what technical test is used in the analysis, there remains the scope to justify conduct (even where it fails the AEC test) by demonstrating that it is objectively justified or produces efficiencies such that the anti-competitive effects (on as-efficient competitors) are outweighed. In *Intel RENV*, the GC likewise distinguished the AEC test from the AEC standard through its treatment of the Commission’s application of the test as a distinct (and not compulsory) assessment of foreclosure capability from the compulsory requirement of analysing foreclosure capability when establishing the conduct’s potential to produce anti-competitive effects.^131^

The AEC test must also be distinguished from an economic, effects-based approach. This is because the viewpoint that *Intel* has limited application because it only applies to pricing conduct or because the Court has held that the AEC test is not necessary or relevant in all cases can engender an interpretation of the existing jurisprudence that the Court has *not* adopted an economic, effects-based approach.^132^ Indeed, when the standard is confused with the test, then the test which requires a price–cost analysis can logically only be applied in, and be relevant to, assessing pricing conduct. Yet, once the AEC test is understood as one way of establishing the effects of a conduct on equally or more efficient competitors, then the inapplicability of that particular test does not imply the inapplicability or irrelevance of an economic, effects-based approach. The possibility of adopting an economic, effects-based approach without necessarily applying the AEC test in all cases is, indeed, the only way to rationalise coherently the recent evolution of the CJEU’s jurisprudence.^133^

## 4. The Hybrid Era

If one were to predict the future jurisprudential evolution based on where the case law currently stands and where it started, that future would likely involve the Court’s progressively mixing the traditional with the modern. This evolving approach provides more certainty and method than the traditional approach to abuse, but it is still in flux. This is because many ‘legacy’ concepts from the traditional era remain firmly in the jurisprudence—‘competition on the merits’ and ‘special responsibility’ of dominant undertakings and references to concepts such as ‘detriment to public interest’ in expressing the function of competition rules may or may not be red herrings.^134^ Many of these ‘legacy’ rules and concepts make awkward bedfellows with an economic, effects-based approach. The CJEU has undoubtedly become more economic in its approach and more demanding of the Commission in that respect, but currently one cannot discern how far the turn of the tide will go. So far, we appear to have entered the *hybrid* era.

Notably, in *Intel*, the CoJ did not go as far as accepting—at least, expressly—AG Wahl’s position that ‘given its economic character, competition law aims, in the final analysis, to enhance efficiency’.^135^ However, ultimately, the Court also did not side with AG Kokott, who had advised the Court not to be influenced by the ‘zeitgeist’ and ephemeral trends (ie the more economic approach) and to adhere to the traditional era principles.^136^ The tension between these two opposing perspectives on the direction of travel for the law on abuse of dominance is clearly reflected in the Court’s recent jurisprudence. A poignant example is that ‘competition on the merits’ is referenced 16 times in a total of 15 pages in *ENEL*, which, as discussed above, adopts an economic, effects-based approach.^137^ Thereafter, in *Unilever*, which also adopts an effects-based approach, the Court remarked that an abuse can be established based on either its effects on as-efficient competitors *or* ‘the use of means other than those which come within the scope of “normal” competition, that is to say, competition on the merits’, implying that ‘competition on the merits’ may constitute an alternative, additional standard to the AEC standard for assessing abuse.^138^ Although this position appears to be abandoned in more recent case law,^139^ it is nevertheless important to note in any case that in *ENEL* (which *Unilever* refers to in support), the CoJ—to this author’s knowledge, for the first time—elaborated on the meaning of the concept of ‘competition on the merits’. The CoJ held that

[a]ny practice the implementation of which holds no economic interest for a dominant undertaking, except that of eliminating competitors so as to enable it subsequently to raise its prices by taking advantage of its monopolistic position, must be regarded as a means other than those which come within the scope of competition on the merits.^140^

This description of what does not constitute competition on the merits is significant because the CoJ did *not* refer to concepts from the traditional era, such as freedom to compete or freedom of choice of supply, but provided an *economic* description. In fact, the CoJ’s understanding of ‘competition on the merits’ is essentially a description of the ‘no economic sense’ test, an economic standard which assesses whether conduct is economically rational in determining whether it is anti-competitive.^141^ The CoJ’s description of competition on the merits can be applied to *all* exclusionary abuses, including pricing and non-pricing conduct. It is also noteworthy that in *ENEL* the CoJ referred to the ‘well-being’ of intermediary and final consumers as the ‘ultimate objective warranting the intervention of competition law in order to penalise abuse of a dominant position’.^142^ Thus, the Court characterised the objective of the prohibition of abuse of dominance in terms of protecting ‘consumer welfare’.^143^ This statement also evidences the jurisprudence’s evolution towards becoming more economic. Yet, interestingly, the Court stipulated the objective of article 102 by integrating another legacy concept—‘effective competition *structure*’—with this economic approach. The Court ultimately mixed its previous, formalistic approach focusing on the protection of a given market structure, which can lead to the protection of competitors for its own sake, with its economic, effects-based approach.^144^ The Court held that it is possible for a competition authority to discharge its burden of proof by showing that the conduct of the dominant undertaking ‘could impair, by using resources or means other than those governing normal competition, an effective competition structure, without it being necessary for that authority to prove that that practice may also cause direct harm to consumers’.^145^ Yet, because the ultimate aim of the prohibition is to protect consumer welfare, the dominant undertaking can ‘nevertheless escape the prohibition’ by showing that the exclusionary effect is counterbalanced or outweighed by positive efficiency gains for consumers.^146^ Thus, the Court used the possibility of an objective justification based on benefits to consumers arising from conduct which is otherwise exclusionary as the means of merging its traditional approach with the modern approach. The lengths to which the Court goes to manage the tug of war between those in favour of and those against an economic, effects-based approach is striking.^147^

Irrespective of the remaining legacy concepts and their role in the evolving jurisprudence, it is difficult to view the recent evolution of the jurisprudence, whereby economic concepts and principles have been integrated into the fundamental principles of establishing an abuse, as a fad. There have simply been too many pronunciations of an economic, effects-based approach in article 102 jurisprudence and too many losses for the Commission over the last decade. Although the trend is certainly reversible given the institutional framework (eg lack of precedent), it is likely to signify a progressive paradigm shift in the CJEU’s jurisprudence whereby the Court becomes more systematic and methodical in its approach to article 102. Thus, the recent evolution of the jurisprudence is possibly a sign of coming of age of the CJEU and the future case law will likely provide more ‘clarifications’ of the legacy concepts and principles and introduce more method—and potentially more economics—into the approach to abuse.

The evolution of the CJEU’s jurisprudence towards a more economic, effects-based approach raises an interesting potential dilemma in digital markets, specifically. This is because the Commission has promulgated an approach focused on *ex ante* regulation in digital markets. The most important outcome of this trend is the DMA which introduces rules applicable to certain large digital platforms (‘gatekeepers’) in certain markets.^148^ The DMA has no scope for effects-based analyses and applies irrespective of the presence or absence of any effects of conduct on competition, competitors or consumers. However, it is well documented that practically all the rules in the DMA are based on antitrust enforcement proceedings, and there is significant overlap between the application of EU competition rules and the DMA.^149^ Moreover, the Commission continues to enforce article 102 in digital markets,^150^ and national competition authorities enforce article 102 but have no powers to enforce the DMA.^151^ Both sets of rules ultimately seek to control the use of market power.^152^ This background raises the possibility of clashes between the treatment of the same practice under article 102 and under the DMA since they both potentially apply to the same conduct when adopted by gatekeepers. Such a clash can lead to a bifurcation of the approach in digital markets, with the implication that similar practices may be treated significantly differently depending on which regime they are caught under. This can happen where one gatekeeper is pursued under article 102 and another is subject to the DMA for conduct which leads to similar *effects* on competition in their respective markets.^153^ Likewise, different legal outcomes may occur in cases with similar facts where article 102 is applied to practices of large platforms in markets in which they are not designated as gatekeepers, but occupy a dominant position. The Commission can, given its stance and the evolution of the CJEU’s jurisprudence, increasingly use DMA powers instead of competition law. Such an approach can undermine the evolution of the case law and the move towards a more effects-based approach under article 102. That situation will only be aggravated if the Commission adopts guidelines on article 102 which do not embrace an effects-based approach to the same extent as the CJEU.^154^ Likewise, given the application of the DMA in digital markets and the CJEU’s effects-based approach, the enforcement of article 102 in non-digital markets (or against non-gatekeeper digital platforms) can take a very different shape, subjecting dominant undertakings in other markets (or of different size) to a substantially disparate approach than digital gatekeepers. Such bifurcation of treatment under article 102 and the DMA raises concerns with respect to legal certainty and equality of treatment, but may be potentially unavoidable given the legal framework established by the DMA, the overlap between the two sets of rules and the divergence of approaches thereunder.

## 5. Conclusion

This article critically inquired into the legal elements of exclusionary abuse under article 102 through an analysis and synthesis of the evolving jurisprudence of the CJEU on the topic. The development of the jurisprudence, divided into the ‘traditional’, ‘modern’ and ‘hybrid’ eras, demonstrated that the law on the prohibition of an abuse of a dominant position has progressively evolved to adopt a more economic, effects-based approach to replace a formalistic approach. In examining the progression of the jurisprudence, this article corrected some fundamental misconceptions, such as the existence of an object–effect dichotomy under article 102 and of different legal tests for pricing and non-pricing conduct. The article also clarified the distinction between the AEC *standard* and the AEC *test*, and identified the legal requirements of abuse. The article established, based on the preponderance of the evolved jurisprudence, that a violation of article 102 cannot be proven without an analysis of the foreclosure capacity of the conduct in question, which means the actual or potential foreclosure effects on competitors at least as efficient as the dominant undertaking, taking into account all relevant circumstances. This article demonstrated that this standard applies to *all* types of exclusionary abuse, irrespective of the form or nature of the contested conduct.

It is notable that, in recent years, the CJEU’s approach to article 102 has become more methodical, more economically informed and more demanding of the Commission regarding the latter’s enforcement practice. This paradigm shift at the CJEU comes at a time when the Commission appears disenchanted with the ‘more economic approach’ which it previously advocated and not so keen on future competition law enforcement in important contexts such as digital markets. The swapping of the positions between the CJEU and the Commission on the adoption of an economic approach means that the two institutions may still be as equidistant to one another as they were when the Commission undertook a reform of its approach to article 102 over a decade ago. This difference of opinions matters significantly in antitrust enforcement and regulatory initiatives in digital markets specifically, and can lead to different approaches to similar practices with similar effects on competition depending on the enforcement context. Such an outcome is undesirable for the coherence of the law, but may be unavoidable given the current legal and institutional frameworks. If that outcome transpires, the slow-but-significant evolution of the law on the prohibition of abuse of a dominant position may be nowhere near as impactful as it would have been a decade ago.

## Footnotes

1 See Elizabeth Warren, ‘Here’s How We Can Break Up Big Tech’ (8 March 2019) <https://medium.com/@teamwarren/heres-how-we-can-break-up-big-tech-9ad9e0da324c>; US President Joseph R Biden Jr, ‘Executive Order on Promoting Competition in the American Economy’ (9 July 2021); US House of Representatives, Subcommittee on Antitrust, Commercial, and Administrative Law, ‘Hearing: Online Platforms and Market Power, Part 6: Examining the Dominance of Amazon, Apple, Facebook, and Google’ (29 July 2020), when the CEOs of Amazon, Apple, Facebook and Google testified under oath at a live session, broadcast to the public <https://www.congress.gov/event/116th-congress/house-event/110883>.

2 On rising market power and concentration and competitive implications, see eg IMF, ‘Rising Corporate Market Power: Emerging Policy Issues’ (2021) Staff Discussion Note SDN21/01; <www.imf.org/-/media/Files/Publications/SDN/2021/English/SDNEA2021001.ashx>.

3 The most prominent example of such regulations is the Digital Markets Act; Regulation (EU) 2022/1925 of the European Parliament and of the Council of 14 September 2022 on contestable and fair markets in the digital sector and amending Directives (EU) 2019/1937 and (EU) 2020/1828 (Digital Markets Act) [2022] OJ L265/1.

4 See eg European Commission, Case COMP/AT.40411—*Google Search (AdSense)*, 20 March 2019, currently on appeal in Case T-334/19 *Google and Alphabet v Commission* [2019] OJ C255/46; European Commission, Case COMP/AT.40099—*Google Android*, 18 July 2018, mostly upheld on appeal in Case T-604/18 *Google and Alphabet v Commission (Google Android)* ECLI:EU:T:2022:541 and currently on further appeal in Case C-738/22 P *Google and Alphabet v European Commission (Google Android)*; Case COMP/AT.39740—*Google Search (Shopping*), 27 June 2017, mostly upheld in Case T-612/17 *Google and Alphabet v Commission (Google Shopping)* ECLI:EU:T:2021:763 and currently on further appeal in Case C-48/22 P *Google and Alphabet v Commission (Google Shopping)* [2022] OJ C191/10.

5 Robert O’Donoghue and Jorge Padilla, *The Law and Economics of Article 102 TFEU* (3rd edn, Hart Publishing 2020) 266.

6 Opinion of AG Rantos in Case C-377/20 *Servizio Elettrico Nazionale, ENEL and Others v Autorità Garante della Concorrenza e del Mercato and Others* (*ENEL*) ECLI:EU:C:2021:998, [53].

7 Pablo Ibanez Colomo, *The Shaping of EU Competition Law* (CUP 2018) 312–13.

8 See, amongst others, Ekaterina Rousseva, *Rethinking Exclusionary Abuses in EU Competition Law* (Hart Publishing 2010); Renato Nazzini, *The Foundations of European Union Competition Law: The Objective and Principles of Competition Law* (OUP 2011); Pınar Akman, *The Concept of Abuse in EU Competition Law: Law and Economic Approaches* (Hart Publishing 2012, reprinted 2015); O’Donoghue and Padilla (n 5).

9 Case C-413/14 P *Intel Corp v European Commission* ECLI:EU:C:2017:632.

10 Commission, ‘Call for Evidence for an Initiative: EU Competition Law—Guidelines on Exclusionary Abuses by Dominant Undertakings’ (27 March 2023) <https://ec.europa.eu/info/law/better-regulation/have-your-say/initiatives/13796-EU-competition-law-guidelines-on-exclusionary-abuses-by-dominant-undertakings_en>; Commission, Press Release, ‘Antitrust: Commission Announces Guidelines on Exclusionary Abuses and Amends Guidance on Enforcement Priorities’ (27 March 2023) <https://ec.europa.eu/commission/presscorner/detail/en/ip_23_1911>. As well as announcing prospective guidelines, the Commission amended its ‘Guidance on the Commission’s enforcement priorities in applying Article 82 of the EC Treaty to abusive exclusionary conduct by dominant undertakings’ [2009] OJ C45/7; see Commission, ‘Amendments to the Communication from the Commission Guidance on the Commission’s enforcement priorities in applying Article 82 of the EC Treaty to abusive exclusionary conduct by dominant undertakings’ (Communication) C(2023) 1923 final <https://competition-policy.ec.europa.eu/document/3c8af31c-1bf0-467a-b4a7-a69da6e722bb_en>.

11 For a discussion, see Akman (n 8) 94–5.

12 See guidance (n 10) [19].

13 See eg ibid [23]–[27].

14 DMA (n 3).

15 art 102(1). As the current article focuses on abuse, the requirements of establishing a dominant position or an effect on trade between Member States are not discussed.

16 On objective justification, see eg Case 27/76 *United Brands Company and United Brands Continentaal BV v Commission* ECLI:EU:C:1978:22, [184], [188]; Case C‑52/09 *Konkurrensverket v TeliaSonera Sverige AB* ECLI:EU:C:2011:83, [31], [75]; Case C-209/10 *Post Danmark A/S v Konkurrenceradet**(Post Danmark I)* EU:C:2012:172, [41]–[42].

17 For the criticism and the Commission’s reaction, see eg Pablo Ibanez Colomo and Andriani Kalintiri, ‘The Evolution of EU Antitrust Policy: 1966–2017’ (2020) 83(2) MLR 321, 334–8; Anne C Witt, ‘The European Court of Justice and the More Economic Approach to EU Competition Law—Is the Tide Turning?’ (2019) 64(2) Antitrust Bull 172, 174–6.

18 The documents instrumental to the move towards a more economic, effects-based approach include: Commission, ‘White Paper on Modernisation of the Rules Implementing Articles 85 and 86 of the EC Treaty’ [1999] OJ C132/1; Commission, ‘Green Paper on Vertical Restraints in EU Competition Policy’ COM (96) 721 final; ‘Guidelines on the Application of Article 81(3) of the Treaty’ [2004] OJ C101/97; Economic Advisory Group on Competition Policy (EAGCP), ‘An Economic Approach to Article 82’ (Brussels 2005); Commission, ‘DG Competition Discussion Paper on the Application of Article 82 of the Treaty to Exclusionary Abuses’ (December 2005); guidance (n 10).

19 See Discussion Paper (n 18); guidance (n 10).

20 For comprehensive studies, see Anne C Witt, *The More Economic Approach to EU Antitrust Law* (Hart Publishing 2016); Bruce Wardhaugh, *Competition, Effects and Predictability: Rule of Law and the Economic Approach to Competition* (Hart Publishing 2020).

21 See similarly EAGCP (n 18).

22 Pablo Ibanez Colomo, ‘Beyond the “More Economics-Based Approach”: A Legal Perspective on Article 102 TFEU Case Law’ (2016) 52 CMLR 709, 710.

23 As Adam Smith noted, ‘People of the same trade seldom meet together, even for merriment and diversion, but the conversation ends in a conspiracy against the public, or in some contrivance to raise prices’: Adam Smith, *An Inquiry into the Nature and Causes of the Wealth of Nations* (Glasgow edn, OUP 1976) vol 1, 145.

24 The debate over the objectives of EU competition law and of art 102 is extensive but falls outside the scope of this work. For a selection, see eg Giorgio Monti, *EC Competition Law* (CUP 2007) 48–52; Pınar Akman, ‘Searching for the Long-Lost Soul of Article 82EC’ (2009) 29(2) OJLS 267; Ariel Ezrachi, ‘Sponge’ (2016) 5(1) JAE 49; Ioannis Lianos, ‘Polycentric Competition Law’ (2018) 71 CLP 161; Konstantinos Stylianou and Marios Iacovides, ‘The Goals of EU Competition Law: A Comprehensive Empirical Investigation’ (2022) 42 LS 620.

25 Protocol (No 27) on the internal market and competition [2008] OJ C115/309. Protocols have the same force as Treaty provisions; Treaty on the European Union (TEU), art 51.

26 See eg the discussion in Monti (n 24) 79–82; O’Donoghue and Padilla (n 5) 89–90.

27 See eg the Legal Service’s resistance against the more economic approach in *Intel*, where it argued on appeal that ‘the Commission disputes that the AEC test forms part of the legal analysis of the contested decision’ and that the Commission ‘contends that, for [fidelity rebates], it is not necessary to establish actual or potential foreclosure effects on a case-by-case basis’; see Case T-268/09 *Intel Corp v Commission* ECLI:EU:T:2014:547, [140] and [71] respectively. Notably, in *Intel*, the Commission had applied the AEC test in line with the more economic approach promulgated in the guidance (n 10); see Commission, Case COMP/C-3/37.990—*Intel* (2009) summary [2009] OJ C227/13 [916], [1002]–[1616] <ec.europa.eu/competition/sectors/ICT/intel_provisional_decision.pdf>. For a discussion of *Intel* and its implications, see the text after n 63.

28 See Wardhaugh (n 20) 109–11, explaining how the Commission did *not* apply the more economic approach in its enforcement practice post-guidance.

29 Compare *Post Danmark I* (n 16), hailing a more economic, effects-based approach, with *Tomra*, decisively adopting a more formalistic approach, delivered three weeks apart (Case C-549/10 P *Tomra Systems ASA and Others v Commission* ECLI:EU:C:2012:221).

30 For the Commission’s ‘staggering’ success rate, see O’Donoghue and Padilla (n 5) 94.

31 Case 85/76 *Hoffmann-La Roche & Co AG v Commission* EU:C:1979:36, [91].

32 The definition of a ‘dominant position’ in the traditional era is similarly not based on relevant economic concepts (eg market power); see *United Brands* (n 16) [65].

33 For references to ‘competition on the merits’, see eg Case 280/08 P *Deutsche Telekom v Commission* (*Deutsche Telekom I*) EU:C:2010:603, [177]; Case C-457/10 *AstraZeneca AB and AstraZeneca plc v Commission* ECLI:EU:C:2012:770, [75].

34 AG Rantos (n 6) [55].

35 ibid [53].

36 Case C-322/81 *NV Nederlandsche Banden Industrie Michelin v Commission* (*Michelin I*) ECLI:EU:C:1983:313, [57]; *Intel* (CoJ) (n 9) [135]; Case C-307/18 *Generics (UK) Ltd and Others v Competition and Markets Authority* ECLI:EU:C:2020:52, [153].

37 See eg *Michelin I* (n 36) [57]; *Deutsche Telekom I* (n 33) [83]; *Intel* (CoJ) (n 9) [135]. On ‘special responsibility’, see Ekaterina Rousseva and Giorgio Monti, ‘The Special Responsibility of Dominant Undertakings’ in Pınar Akman, Or Brook and Konstantinos Stylianou (eds), *Research Handbook on Abuse of Dominance and Monopolization* (Edward Elgar 2023).

38 Similarly, see Richard Whish and David Bailey, *Competition Law* (10th edn, OUP 2021) 197.

39 See eg Pablo Ibanez Colomo, ‘Anticompetitive Effects in EU Competition Law’ (2020) 17(2) Journal of Competition Law & Economics 309, 350.

40 This distinction in art 101 is found in the text of the provision, which prohibits ‘all agreements between undertakings … which have as their object or effect the prevention, restriction or distortion of competition’. For the argument that this dichotomy was introduced into art 102 post-2009 (ie post-guidance), see Wardhaugh (n 20) 112. cf Ibanez Colomo (n 22) 712–13, positing that some practices are *prima facie* prohibited as abusive irrespective of their effect on competition (and have always been so). cf O’Donoghue and Padilla (n 5) 278.

41
*Deutsche Telekom I* (n 33) [234]; *TeliaSonera* (n 16) [61]–[63].

42 Case C-7/97 *Oscar Bronner GmbH & Co KG v Mediaprint Zeitungs- und Zeitschriftenverlag GmbH & Co KG and others* ECLI:EU:C:1998:569, [41].

43 See the discussion in O’Donoghue and Padilla (n 5) 270.

44 Case C-62/86 *AKZO Chemie BV v Commission* ECLI:EU:C:1991:286, [71]. See also Case C-202/07 P *France Télécom SA v Commission* ECLI:EU:C:2009:214, [109]–[114]. For the relevance of recoupment to the economic theory of harm, see O’Donoghue and Padilla (n 5) 382.

45
*Michelin I* (n 36) [73].

46 Case C-95/04 P *British Airways plc v Commission* ECLI:EU:C:2007:166, [61].

47 See text to n 69.

48 Case T-203/01 *Manufacture française des pneumatiques Michelin v Commission* (*Michelin II*) ECLI:EU:T:2003:250, [241] (citation omitted).

49 ibid [237].

50 Case T-228/97 *Irish Sugar plc v EC Commission* ECLI:EU:T:1999:246, [170].

51 As noted by the GC, the Commission decision assessed both. However, the ‘effect’ that is relevant to the Court’s discussion (and what the cited paragraph of the Commission decision refers to) is an ‘effect on trade between Member States’, and *not* the effects of a practice on competition. Thus, the GC reference in *Michelin II* (n 48) to its ruling in *Irish Sugar* (n 50) regarding the distinction between anti-competitive object and effect is highly contentious since its source reference refers to a different type of effect than its statement in *Michelin II*. Having said that, in a small number of subsequent rulings, the GC repeated its pronouncement equating object and effect in upholding Commission findings that an effects analysis is not necessary; see Case T‑340/03 *France Télécom v Commission* [2007] ECR II‑107, [195]; *Intel* (GC) (n 27) [199], [203], annulled on appeal in *Intel* (CoJ) (n 9). The type of practices to which this line of jurisprudence is applicable has been dubbed ‘naked restrictions’. On whether they are by-object violations, see n 105.

52
*Expressio unius est exclusio alterius* (the inclusion of one is the exclusion of others) dictates that the drafters must have considered the dichotomy under art 102, too, but rejected its inclusion thereunder. On statutory interpretation and *expressio unius*, see Larry M Eig, ‘Statutory Interpretation: General Principles and Recent Trends’, Congressional Research Service Report (2014) 17–19 <https://sgp.fas.org/crs/misc/97-589.pdf>.

53 See text to n 103.

54 This is unlike the case under art 101, where the Court’s jurisprudence cannot abolish the category of by-object restrictions because the distinction is enshrined in the Treaty.

55 This is acknowledged by the Commission (n 10); see Linsey McCallum and others, ‘A Dynamic and Workable Effects-Based Approach to Abuse of Dominance’ (European Commission 2023) Competition Policy Brief No 1/2023, 4. Reining in the CoJ is, arguably, the motivation behind the prospective guidelines.

56 See Ekaterina Rousseva and Mel Marquis, ‘Hell Freezes Over: A Climate Change for Assessing Exclusionary Conduct under Article 102 TFEU’ (2013) 4(1) Journal of European Competition Law & Practice 32, 32, also identifying *Post Danmark I* (n 16) (delivered in 2012) as the critical ruling regarding the jurisprudential shift. Others identify *Deutsche Telekom I* (n 33) (delivered in 2010) as marking the start of the shift; see eg Witt (n 17) 197–8; Ibanez Colomo (n 22) 713.

57
*Post Danmark I* (n 16) [22].

58 ibid [25].

59
*Deutsche Telekom I* (n 33) [177]. See also *TeliaSonera* (n 16) [31]–[33], [39]–[40], [43], [63]–[64], [67], [70], [73]; *Post Danmark I* (n 16) [21], [22], [25], [38].

60 See eg *Deutsche Telekom I* (n 33) [250], [252], [253], [254], [259]; *TeliaSonera* (n 16) [64]; *Post Danmark I* (n 16) [25], [40], [44]; Case C-23/14 *Post Danmark A/S v Konkurrencerådet* (*Post Danmark II)* ECLI:EU:C:2015:651, [46], [47], [48], [49]. Although the jurisprudence could be more systematic and clearer regarding this, phrases such as ‘potential’ and ‘likely’ tend to refer to the same notion in the context of effects, namely the opposite of *actual* effects; see eg *Post Danmark II* [66]–[69]; *TeliaSonera* [64]–[67].

61
*Post Danmark II* (n 60) [64], [65]; *Google (Search) Shopping* (GC) (n 4) [457]. See also Commission Decision, Case AT.40220—*Qualcomm* (Exclusivity Payments) (2018) [381] <https://ec.europa.eu/competition/antitrust/cases/dec_docs/40220/40220_2702_4.pdf>. *Qualcomm* was annulled on appeal in Case T-235/18 *Qualcomm v Commission* ECLI:EU:T:2022:358 and is discussed in the text around n 106.

62 For a discussion of the distinction, see the text around n 117.

63 Witt (n 17) 208. The literature on *Intel* well demonstrates the divide between commentators regarding whether the repudiation of the effects-based approach in the GC’s ruling was a ‘good’ or ‘bad’ development; see eg Wouter PJ Wils, ‘The Judgment of the EU General Court in Intel and the So-Called “More Economic Approach” to Abuse of Dominance’ (2014) 37(4) World Competition 405, 418, 437; Paul Nihoul, ‘The Ruling of the General Court in Intel: Towards the End of an Effect-based Approach in European Competition Law?’ (2014) 5(8) Journal of European Competition Law & Practice 521, 530; Luc Peeperkorn, ‘Conditional Pricing: Why the General Court Is Wrong in Intel (and What the Court of Justice Can Do to Rebalance the Assessment of Rebates)’ [2015] (1) Concurrences 43, 43–4.

64
*Intel* (CoJ) (n 9) [133]–[134].

65
*Intel* (GC) (n 27).

66 For the Commission’s record in appeals, see n 30.

67
*Intel* (CoJ) (n 9) [138].

68 O’Donoghue and Padilla (n 5) 547.

69
*Intel* (CoJ) (n 9) [138].

70 ibid [139].

71 ibid [140].

72 Further evidencing the jurisprudential evolution, commentators questioned after *Post Danmark I* (n 16) whether the AEC test applied only to some price-based abuses or to all price-based abuses (eg also to rebates); see Rousseva and Marquis (n 56) 45. Between *Post Danmark I* and *Intel*, the pertinent question appears to have become whether the AEC test applied to all price-based exclusionary abuses or to *all* exclusionary abuses.

73 See James S Venit, ‘The Judgment of the European Court of Justice in Intel v Commission: A Procedural Answer to a Substantive Question?’ (2017) 13 ECJ 172.

74 See text to n 69 and text to n 82. See also O’Donoghue and Padilla (n 5) 547, 561.

75 See eg Witt (n 17) 209.

76 See *Intel* (CoJ) (n 9) [138], which reads: ‘However, that case-law must be further clarified’. ‘That case law’ is found in the previous paragraph, where the Court mentions two different types of practices, namely exclusive dealing (with or without rebates) and loyalty rebates; ibid [137]. Given that exclusive dealing does not necessarily involve a pricing element and given that more than one practice is mentioned in [137] to which [138] refers when ‘clarifying’ the case law, positing that the ‘clarification’ of the jurisprudence pertains only to rebates or only to pricing practices is unduly restrictive.

77 See text around n 85.

78 See text around n 70.

79
*Intel* (CoJ) (n 9) [140] (emphasis added).

80 ibid (emphasis added).

81 ibid [141].

82 ibid.

83 ibid [149] (emphasis added). An appeal to the CoJ is on points of law only (and does not involve a review of *facts*); TFEU, art 256.

84 See eg Case T-814/17 *Lietuvos geležinkeliai AB v Commission (Lithuanian Railways*) ECLI:EU:T:2020:545, [76]; Case T-827/14 *Deutsche Telekom AG v Commission* ECLI:EU:T:2018:930, [87]. See also *MEO*, where the CoJ transposes the principles from *Intel* even to cases of exploitative abuse, augmenting the importance and generality of the approach in *Intel* for *all* abuse cases; Case C-525/16 *MEO—Servicos de Comunicacoes e Multimedia SA v Autoridade de Concorrencia* ECLI:EU:C:2018:270, [31].

85
*Generics* (n 36) [154] (emphasis added) (citations omitted), with reference to *TeliaSonera* (n 16) [64]–[66], [68]; *Intel* (CoJ) (n 9) [138]. See also *Generics* [157], [167] for references to the necessity and relevance of *effects*.

86 Case C-165/19 P *Slovak Telekom v Commission* ECLI:EU:C:2021:239, [51]. It is clear from the judgment that the relevant effects are those on as-efficient competitors [109].

87 Case C-377/20 *Servizio Elettrico Nazionale, ENEL and Others v Autorità Garante della Concorrenza e del Mercato and Others* ECLI:EU:C:2022:379, [72].

88 ibid [70].

89 On the implications of the distinction between the AEC *standard* and the AEC *test*, see the text around n 117.

90
*ENEL* (n 87) [79].

91 Case C-680/20 *Unilever Italia Mkt Operations Srl v Autorita Garante della Concorrenza e del Mercato* ECLI:EU:C:2023:33, [50], [52], [59].

92 ibid [49], read in conjunction with [50].

93
*Google (Search) Shopping* (GC) (n 4) [441]. See also [518]. The GC partially annulled the Commission’s decision in *Google Shopping* because the Commission failed to demonstrate that the conduct ‘has had—at least potential—anticompetitive effects in the relevant market or markets’; [459]. See also [438].

94 ibid [435].

95 ibid [438].

96
*Unilever* (n 91) [52], [53]. See also [62], which holds that the competition authority ‘is required, in order to find an abuse of a dominant position’ to establish that the conduct is ‘capable of restricting competition’.

97 See Case T-286/09 RENV *Intel Corp v Commission* ECLI:EU:T:2022:19, [507], [512], [521].

98 ibid [521].

99 ibid [518], [524].

100 In its appeal against *Intel RENV*, the Commission argues that the GC misinterpreted the CoJ’s guidance; see Appeal by European Commission on 5 April 2022 against the judgment of the General Court (Fourth Chamber, Extended Composition) delivered on 26 January 2022 in Case T-286/09 RENV, *Intel Corporation v Commission* (Case C-240/22 P) [2022] OJ C222/21.

101
*Intel RENV* (n 97). This is so except for the part of the decision concerning ‘naked restrictions’ (which involved Intel’s making payments to Original Equipment Manufacturers to delay, cancel or restrict the launch of products with AMD chips); [527], [529], [530]. The GC, however, annulled the entire fine. The Commission has appealed *Intel RENV*; see Case C-240/22 P *Commission v Intel Corporation* (pending). The Commission has also reimposed a fine for ‘naked restrictions’, which Intel has appealed; see Commission, Case AT.37990—*Intel*, 22 September 2023 and Case T-1129/23 *Intel Corporation v Commission* (pending), respectively.

102
*Intel RENV* (n 97) [526] (emphasis added).

103 ibid [522].

104
*Unilever* (n 91) [51], [52]. For the argument that exclusive dealing is *prima facie* prohibited as abusive, see eg Ibanez Colomo (n 22) 712, 714, 722. The CoJ’s recent reference to ‘object’ in Case C-333/21 *European Superleague Company v FIFA and UEFA* ECLI:EU:C:2023:1011, [131] when discussing art 102 is unfortunate and should be clarified in future rulings. However, a careful reading of the judgment suggests that the reference to ‘object’ does not undermine the position that an object–effect dichotomy such as the one in art 101 does not exist in art 102. This is because at [131] the CoJ appears to refer *not* to the concept of an anti-competitive object, which is to be distinguished from an anti-competitive effect, but to the specific object of *preventing* the *entry* of potential competitors, which the Court distinguishes from actual or potential *exclusion* of rivals. The fact that the Court does not use ‘object’ in the same sense as in art 101 is also demonstrated by the fact that at [204] the Court stipulates that the dominant undertaking can justify its conduct by demonstrating that efficiency gains from the conduct counteract the ‘likely harmful effects’ of that conduct on ‘competition and consumer welfare’. If the Court tried to endorse an object–effect dichotomy at [131], then [204] of the same judgment could not make any sense since, first, the dominant undertaking would be expected to rebut a position which the Commission (who bears the burden of proof) did not establish in having to prove that efficiencies offset ‘likely harmful effects’ (which the Commission would not have needed to demonstrate for a by-object violation), and second, by-object violations cannot be disproved by demonstrating lack of harmful effects. Finally, the Court’s reference at [185] to conduct which ‘by its nature’ infringes art 102 is ambiguous, because the discussion and case law at [183]–[185] appear relevant to art 101, rather than art 102.

105 See also AG Rantos (n 6) [55]. This position must also apply to ‘naked restrictions’, which refer to findings of abuse where the conduct ‘can only raise obstacles to competition’ without creating any efficiencies so that its anti-competitive effects may be *inferred*; see guidance (n 10) [22]. Naked restrictions are not by-object violations: the possibility of demonstrating that the contested practice cannot have foreclosure effects on as-efficient competitors applies to *all* exclusionary abuses. For by-object violations under art 101, it is not possible under any circumstances to disprove a violation by demonstrating the absence of effects, meaning that any presumption of abuse which may exist for ‘naked restrictions’ is conceptually wholly distinct from the approach to by-object violations. The possibility of disproving a by-object violation by demonstrating absence of effects would annihilate the by-object framework. Proving the absence of foreclosure effects to rebut a finding of abuse is not merely a theoretical possibility and can be demonstrated, for example, by proving that the competitor could not have entered the market even in the absence of the dominant undertaking’s abusive conduct; see eg Case T-136/19 *Bulgarian Energy Holding et al v Commission* ECLI:EU:T:2023:669, [952]–[953].

106
*Qualcomm* (GC) (n 61). The Commission has not appealed. More recently, another Commission decision was annulled in *BEH* (n 105).

107
*Qualcomm* (GC) (n 61) [344]–[345], [368], [411], [415], [417], [450], [462], [505], [507]–[511].

108 ibid [354].

109 ibid [355].

110 ibid [356].

111 ibid [354] refers to the Commission’s having to carry out an analysis of the foreclosure capacity of competitors which are at least as efficient as the dominant undertaking. Thus, it appears that [356], which refers to the Commission’s conducting ‘such an analysis’, should have been [355] and not [356]. The problem is that it is unclear whether [356] genuinely aims to restrict the scope of that statement in [355] to cases where the undertaking challenges the Commission’s allegations and the Commission undertakes an AEC analysis, but it is obvious that [356] is misplaced since its content does not follow from the previous paragraph as intended by the Court. Recently, the GC applied the *Intel* principles to annul partially a decision for the Commission’s failure to establish the exclusionary effects of the conduct on as-efficient competitors; *Google Android* (GC) (n 4) [795]–[802].

112 See *Qualcomm* (GC) (n 61) [424].

113 Contrast with the commentary discussed at text to n 75 and with the guidance (n 12).

114 ‘Foreclosure effects’ include *prevention* of *entry* of potential competitors; see *ESC* (n 104) [131].

115 See eg *Generics* (n 36) [154]; *Qualcomm* (GC) (n 61) [355]; *ENEL* (n 87) [72]; *Google (Search) Shopping* (GC) (n 4) [441].

116 cf Ibanez Colomo (n 39) 321; Rousseva and Marquis (n 56) 45; Witt (n 17) 194. The finding that there are no by-object violations under art 102 does not exclude the possibility of adopting presumptions (see eg text to n 44) provided that the presumptions are rebuttable based on (the absence of actual or likely) *effects*.

117 See eg Rousseva and Marquis (n 56) 45; Witt (n 17) 209. For the use of ‘test’ and ‘standard’ interchangeably, see Ioannis Lianos, ‘The Price/Non Price Exclusionary Abuses Dichotomy: A Critical Appraisal’ (2009) 2 Concurrences. See also O’Donoghue and Padilla (n 5) 283.

118 cf Ibanez Colomo (n 7) 35, distinguishing *standards* from *rules* and arguing that, under art 102, some practices are subject to a standard and some to a rule.

119 See Richard A Posner, *Antitrust Law* (2nd edn, University of Chicago Press 2001) 194–5.

120 See ibid 195.

121 ibid 196.

122 ibid 196, 197.

123 See eg guidance (n 10) [23], [25], [26], [27]. See also *ENEL* (n 87) [78], [79]; *Slovak Telekom* (n 86) [110].

124 See similarly Whish and Bailey (n 38) 753.

125 In *Google (Search) Shopping* (GC) (n 4), the GC *both* adopts the *Intel* framework by requiring a demonstration that conduct must have at least potential anti-competitive effects to constitute abuse ([439]–[440]) *and* rejects Google’s argument that the Commission should have demonstrated that the competitors which were allegedly excluded from the market were as efficient as Google, by iterating that the use of the AEC test was not warranted in the case because the relevant practices were not pricing practices ([538]). Thus, the Court confuses the standard (ie whether, as a matter of principle, the conduct can exclude from the market competitors as efficient as the dominant undertaking) with the technical test.

126
*ENEL* (n 87) [79], provided in full at text to n 90.

127 ibid [80], [83]. *Bronner* does not establish a general approach to abuse. However, *Bronner* (n 42) [44]–[45] (to which the CoJ refers in *ENEL* (n 87) [83] in relation to non-pricing conduct), read alongside *Bronner* [46], essentially establish that the relevant question is whether as-efficient competitors can replicate the input to which access is sought when determining the indispensability of access.

128 See *ENEL* (n 87) [101], where the Court remarks regarding the contested practice that ‘such conduct would necessarily be incapable of being adopted by a hypothetical as-efficient competitor’.

129 See also *Unilever* (n 91) [39], [52], which in essence concern the *standard*, and [56] *et seq*, which discuss the *test*. In *Unilever* [59], the CoJ confirmed the relevance of the AEC *test* even for *non-pricing* conduct.

130 See *Intel* (CoJ) (n 9) [139]–[140].

131 See *Intel RENV* (n 97) [518], [522], [525]. The Commission ‘must’ analyse the foreclosure capability of the conduct; see eg [492], [507], [522]; *Intel* (CoJ) (n 9) [139]. Clearly, the GC deems the AEC *test* as one possible *quantitative* approach to demonstrating a conduct’s anti-competitive capability; *Intel RENV* [477]. Similarly, see *ENEL* (n 87) [81]; *Post Danmark II* (n 60) [57]; *Unilever* (n 91) [57].

132 See eg Nihoul (n 63) 531, writing after *Intel* (GC) (n 27) and suggesting that the CoJ’s upholding of the ruling would imply the end of the more economic approach to art 102.

133 See eg *Post Danmark II* (n 60) [57]; *ENEL* (n 87) [81], where the CoJ stipulates that the AEC test is not necessary to establish abuse. See also *Google Android* (GC) (n 4) [641], [643]. If the ‘test’ is confused with the ‘standard’, the statements of the CJEU can be misconstrued to conclude that the AEC *standard* is not necessary or relevant to establishing abuse.

134 See eg *ENEL* (n 87) [41]; *Slovak Telekom* (n 86) [40]; *Intel RENV* (n 97) [116]; *Unilever* (n 91) [36].

135 Opinion of AG Wahl in Case C-413/14 P *Intel Corp v Commission* ECLI:EU:C:2016:788, [41].

136 Opinion of AG Kokott in Case C-23/14 *Post Danmark A/S v Konkurrencerådet* (*Post Danmark II*) ECLI:EU:C:2015:343, [4].

137
*ENEL* (n 87). See also *Unilever* (n 91) [39].

138
*Unilever* (n 91) [39].

139 See *ESC* (n 104) [129], [130].

140
*ENEL* (n 87) [77]. Regarding ‘competition on the merits’, *Unilever* (n 91) [39] refers to *ENEL* [71], [75], [76] which directly refer to as-efficient competitors when explaining which type of practice falls outside ‘competition on the merits’.

141 The ‘no economic sense test’ asks whether the conduct would make sense for the dominant undertaking but for its tendency to eliminate or lessen competition; O’Donoghue and Padilla (n 5) 282.

142
*ENEL* (n 87) [46].

143 This concept of ‘consumer welfare’ is better characterised as ‘customer welfare’ since it includes the welfare of intermediary and final consumers; see *ENEL* (n 87) [46]. On the implications of the distinction, see Pınar Akman, ‘“Consumer” versus “Customer”: The Devil in the Detail’ (2010) 37(2) JLS 315.

144 The protection of an ‘effective competition structure’ has long been in the jurisprudence and indicates a formalistic approach given its focus on the *structure* of competition, irrespective of the outcomes in the market (eg lower prices, higher output). See eg *Hoffmann-La Roche* (n 31) [91]; *AKZO* (n 44) [69]; *Post Danmark II* (n 60) [26]; *AstraZeneca* (n 33) [74].

145
*ENEL* (n 87) [47].

146 ibid [46], [47].

147 The tug of war is aptly demonstrated by the Opinions of AG Wahl and AG Kokott; see the text to n 135 and n 136, respectively.

148 See DMA (n 3) arts 3, 5, 6, 7.

149 See eg OECD, ‘Competition Enforcement and Regulatory Alternatives’, OECD Competition Committee Discussion Paper (2021) 31–2 <www.oecd.org/daf/competition/competition-enforcement-and-regulatory-alternatives-2021.pdf>; Pınar Akman, ‘Regulating Competition in Digital Platform Markets: A Critical Assessment of the Framework and Approach of the EU Digital Markets Act’ (2022) 47(1) ELR 85, 94–6.

150 For some recent examples, see n 4.

151 See DMA (n 3) Recital 91. As the DMA applies without prejudice to arts 101 and 102 (DMA, art 1(6)), national competition authorities can (and where applicable *must*) enforce EU competition law, including art 102, against gatekeepers. See Council Regulation (EC) No 1/2003 of 16 December 2002 on the implementation of the rules on competition laid down in Articles 81 and 82 of the Treaty [2003] OJ L1/1, art 3.

152 DMA (n 3) Recital 11 stipulates that it pursues a different (but complementary) objective to competition law. However, given its content, the DMA is arguably a ‘sector-specific competition law’; see Nicolas Petit, ‘The Proposed Digital Markets Act (DMA): A Legal and Policy Review’ (2021) 12(7) Journal of European Competition Law & Practice 529, 529.

153 This can happen where Platform A is designated as a ‘gatekeeper’ and subjected to the DMA rules in Market A, whilst Platform B is not designated as a gatekeeper for Market A (but is a gatekeeper in Market B) and adopts the same practice as Platform A in Market A. Although (and because) Platform B is not technically a gatekeeper in Market A, its conduct could be subject to art 102 if it occupies a dominant position in *a* relevant market. This is a possibility since the concepts of ‘gatekeeper’ and ‘dominant position’ necessitate different assessments under different legal frameworks; see DMA (n 3) Recital 5.

154 This may be the intention with the guidelines. See eg the concern that the ‘move towards an effects-based enforcement of Article 102 raises the question of whether the heightened substantive legal standard that the Union Courts have accorded to it may inadvertently lead to undesirable outcomes’ since ‘an overly rigid implementation of the effects-based approach could set the bar for intervention’ so high that enforcement would become ‘unduly burdensome or even impossible’; McCallum and others (n 55) 4. See also 4–5 discussing the importance of protecting less-efficient competitors under certain circumstances, a position which would clash with the CJEU’s position were it to imply that the AEC *standard* as opposed to the AEC *test* is not always relevant (which it indeed appears to imply, given the separate discussion of the AEC *test* at 6–7). Regrettably, the case law references which the Commission provides to support its said position involve the CJEU’s discussions of the AEC *test*, not the AEC *standard*; thus, it is arguable whether its position has any support in the CJEU’s modern jurisprudence.

